# An integrated quantitative structure and mechanism of action-activity relationship model of human serum albumin binding

**DOI:** 10.1186/s13321-019-0359-2

**Published:** 2019-06-06

**Authors:** Angela Serra, Serli Önlü, Pietro Coretto, Dario Greco

**Affiliations:** 1 Faculty of Medicine and Health Technology, Tampere University, Arvo Ylpön katu 34, Tampere, Finland; 2 DISES, STATLAB, University of Salerno, Giovanni Paolo II 132, Fisciano, Italy; 3 Institute of Biotechnology, University of Helsinki, Finland, Helsinki, Finland; 4 Present Address: Corporate Product Safety/Henkel AG & Co. KGaA, Düsseldorf, Germany; 5 BioMediTech institute, Tampere University, Tampere, Finland

**Keywords:** QSAR, MOA, QSMARt, Molecular descriptors, Human serum albumin binding, Integrative analysis, Safe-by-design, Lasso, Regression

## Abstract

**Background:**

Traditional quantitative structure-activity relationship models usually neglect the molecular alterations happening in the exposed systems (the mechanism of action, MOA), that mediate between structural properties of compounds and phenotypic effects of an exposure.

**Results:**

Here, we propose a computational strategy that integrates molecular descriptors and MOA information to better explain the mechanisms underlying biological endpoints of interest. By applying our methodology, we obtained a statistically robust and validated model to predict the binding affinity to human serum albumin. Our model is also able to provide new venues for the interpretation of the chemical-biological interactions.

**Conclusion:**

Our observations suggest that integrated quantitative models of structural and MOA-activity relationships are promising complementary tools in the arsenal of strategies aiming at developing new safe- and useful-by-design compounds.

**Electronic supplementary material:**

The online version of this article (10.1186/s13321-019-0359-2) contains supplementary material, which is available to authorized users.

## Introduction

Quantitative structure-activity relationship (QSAR) models are increasingly applied in various fields, such as toxicity assessment and drug design [1]. QSAR models developed and validated in line with the Organization for Economic Co-Operation and Development (OECD) criteria [2] are recognized in silico tools for providing reliable activity data, bypassing long and laborious experimental assays. On the basis that structurally similar molecules have similar biological activities, classical QSAR models attempt to predict activity as a function of structural properties numerically defined as molecular descriptors (MDs) [1, 3]. MDs provide extensive chemical information, such as presence and count of different sub-structures, functional groups, connectivity between atoms, topological and geometrical characteristics, which are relevant for predictive studies. Furthermore, 3D alignment-free molecular descriptors, based on two, three and four linear algebraic forms have been introduced to codify novel and orthogonal chemical information [4, 5].

Traditional QSAR models usually neglect the primary biological fingerprint of the exposure, consisting of the ensemble of molecular alterations happening at various cellular compartments of the exposed biological system, hereafter denoted as the mechanism of action (MOA). However, the relationship between structural properties and phenotypic effects of an exposure is indirectly mediated by its MOA. Systematically integrating MOA information, such as gene expression or external bioassay data, into QSAR modelling would expand our understanding of the chemical-biological interactions, hence paving the way to the development of the next generations of safe- and useful-by-design compounds [6, 7].

In the recent years, the implementation of omics technologies in toxicology studies has ignited the new field of toxicogenomics [8]. In this context, in depth molecular profiling opened new possibilities to outline the biosignature or MOA of exposures at an unprecedented granularity. However, to date, this information has been seldom utilized in combination with structural properties of the compounds to predict their effects [9–11].

Indeed, Li et al developed a methodology that jointly analyzes the chemical structural information and the gene expression profiles of cells treated by drugs. By means of a clustering methodology, they identified the most structurally similar sets of chemicals and the minimum set of genes related to chemical structural features [9]. Low et al. [10] used a machine learning methodology based on multiple nonlinear classifiers that integrates chemical descriptors and toxicogenomic data to classify drug molecules based on their hepatotoxicity (toxic/or non-toxic) effect in rats. Perualila-Tan et al. [11] proposed a statistical methodology that combines transcriptomic data and chemical information to predict a biological response by means of gene expression and infer if the response is caused by the presence or absence of a particular chemical sub-structure. These approaches are limited to binary classification problems (toxic/non toxic) and to the identification of correlations between MDs and MOA features. However, when modelling a continuous response variable, integrative regression models are a preferred option. Between the wide range of linear and nonlinear regression models, Lasso based methods have the advantage to generate easy to interpret models, since they automatically perform feature selection and have less parameter to be estimated as compared to nonlinear models, such as random forests, support vector regressors or neural networks.

Here, considering the OECD criteria [2], we propose a computational approach that combines MDs and MOA information to develop integrated quantitative structure and mechanism of action-activity relationship (QSMARt) models with the potential to better explain the role of specific structural properties in a bio-mechanistic way. To the best of our knowledge, the present study is the first report on an integrated QSMARt model to predict the binding affinity to HSA.

## Materials and methods

### Dataset preparation 

Curated experimental binding affinity data of drug and drug-like molecules to HSA ($logK_{HSA}$; the binding constant obtained from the retention time on an immobilized HSA column using affinity chromatography) were obtained from [12]. All structures (as 3D SDF files) were retrieved from PubChem [13] and processed by the software DRAGON v. 7.0 [14] for the calculation of 5,325 MDs. An unsupervised feature reduction was applied to filter the constant ($> 80\%$) and highly intercorrelated descriptors (pairwise correlation among all pairs of descriptors $> 95\%$) prior to training/test set splitting, and variable selection [15]. Thus, a data matrix comprising 1,198 MDs was generated (hereafter denoted as A). Transcriptomic data for drug treatments were retrieved from the Connectivity Map (CMap) build v2.0 repository [16]. Three human cell lines were available in the CMap project: prostate cancer (PC3), breast cancer (MCF7), and leukemia (HL60), respectively. The transcriptomic datasets were analyzed independently for each cell line. Raw data was imported into R v. 3.4 by using the justRMA function from the Bioconductor utilities [17] to annotate probes to Ensembl genes (by using the hthgu133ahsensgcdf (v. 22.0.0) annotation file from the brainarray website http://brainarray.mbni.med.umich.edu/), and to quantile normalize the resulting expression matrix. Next, the experimental batch effect due to technical variables was estimated and removed using the ComBat algorithm implemented in the sva package [18]. Linear models followed by eBayes pairwise comparisons [19] were performed to compute the log fold-change of each gene in each drug-control pairs. Of the 88 chemicals in the curated dataset [12], 59 were identified with reported gene expression data for at least two cell lines of the CMap dataset (MCF7 and PC3). The list of drugs used in this analysis is available in Additional file 1. Consequently, two data matrices of log-fold changes for 11,868 genes in MCF7 (hereafter denoted as B) and PC3 cell lines (hereafter denoted as C) were generated, respectively. Finally, MDs (A) and gene expression profiles (B and C) were collated to create a single dataset (hereafter denoted as X) of 59 drugs and 24,934 features (1198 MDs and 11,868 genes for each cell line) for modeling the $logK_{HSA}$.

### Modeling and validation

QSMARt modeling was performed based on the lasso method [20] and power transformation of the MDs ($\alpha$) and genes ($\gamma$), respectively. 20% of the dataset X was kept as the test set and not used in the model selection phase. The remaining 80% of the data (training set) was further split 100 times in random training (90%) and validation (10%) sets by using a random split validation algorithm (RSVA). The splitting was performed based on the y-response variable, which was divided into three bins, from which the compounds are randomly assigned to train or test sets. Detailed methodology available as in Additional file 2. R scripts are available as Additional file 3. Next, the lasso method is used to fit a linear model to the training set for 100 different values of the lasso penalty estimated from the training matrix [21]. The lasso penalization value leading to the smallest mean squared error (MSE, $\lambda =0.166$), was considered (Additional file 4: Fig. S1). Only the features (MDs and/or genes) with non-zero coefficients were selected to derive the final model. Once the optimal features and parameters were identified, the entire training set was used to build the final model and the test set was only then used for external validation.

The following model was considered to predict the $logK_{HSA}$:

1 $$\begin{aligned} y = X(\alpha , \gamma )\beta + \epsilon \end{aligned}$$

where, $X(\alpha ,\gamma )$ is the matrix obtained by binding the matrices of $A(\alpha )$, $B(\gamma )$ , and $C(\gamma )$, $A(\alpha ) = (|a_{ij}|^{\alpha })$, $B(\gamma ) = (|b_{ij}|^\gamma )$, $C(\gamma ) = (|c_{ij} |^\gamma )$ (for $\alpha >0$ and $\gamma >0$), $\beta$ is the vector of coefficients, and $\epsilon$ is the stochastic error, respectively.

The same power transformation ($\gamma$) was used both for the MCF7 (B) and PC3 cell line (C). Considering $\alpha$ and $\gamma$ fixed, and $\beta$ the only structural/genomic parameter to be estimated, is conceptually equivalent to replacing the original sample measurements *X* with $X(\alpha ,\gamma )$. For fixed $\alpha$ and $\gamma$, the following lasso-type estimator is considered:

2 $$\begin{aligned} {\hat{\beta }}= argmin|| y - X(\alpha , \gamma )\beta ||^{2}_{2} + \lambda ||\beta ||_1 \end{aligned}$$

where $|| \cdot ||_2$ is the euclidean norm, $||\cdot ||_1$ is the $l^1$ norm and $\lambda$ is the lasso penalty.

The parameters $(\alpha ,\beta , \gamma )$ were tuned to minimize the MSE on the training set. The RSVA was performed for a grid of nine distinct $\alpha$ and $\gamma$ values ($\alpha ,\gamma ={0.1,0.25,0.50,0.75,1,1.25,1.50,1.75,2}$) for all 81 possible pairs of $(\alpha _i, \gamma _i)$ with $i=1, \ldots , 81$. For each of the 81 combinations, the relevant set of features $f_t = \beta (\alpha _t,\gamma _t)$ (at $t=1,2,\ldots ,81)$ associated with non-zero coefficients was identified, validated the 60th percentile values of the distributions of the internal metrics computed on the multiple splits were considered), and used to train models on the whole training set. Next, the generated models were used to predict the $logK_{HSA}$ on the test set. Following these steps, a population of candidate models was generated. Goodness of fit, robustness, and predictive performance of the candidate models were evaluated based on up-to-date internal and external validation parameters and criteria (Additional file 1) [22–28].

### Comparison with single view models

In order to validate the QSMARt model, the same procedure was applied to the MDs and MOA features separately. The RSVA procedure was performed on nine $\alpha ={0.1,0.25,0.50,0.75,1,1.25,1.50,1.75,2}$ values for the MDs and nine $\gamma ={0.1,0.25,0.50,0.75,1,1.25,1.50,1.75,2}$ values for the MOA features. Furthermore, these two parameters, together with the $\lambda$ penalty, value were optimized independently for the MDs and MOA to identify the optimal setup that minimizes the MSE on the training set. These analyses led to 9 models for the MDs and 9 models for the MOA features. For each model, the relevant set of features, associated with non-zero coefficients, was identified and validated with the same approach described before. Goodness of fit, robustness, and predictive performance of the candidate models were evaluated based on up-to-date internal and external validation parameters and criteria (Additional file 1) [22–28]. In particular, distributions of the internal validation metrics computed with the RSVA procedure with 100 random splits were compared to identify which model overall give the better predictive performances.

### Applicability domain

Based on the idea of consensus decision[29], different approaches were used to compute the applicability domain (AD) of the identified models. In particular, AD was computed by means of the leverage method [30], the standardization approach [31], the euclidean [32] and city block distance methods [33], and the k-nearest neighbours method [34].

In the leverage method, the response outliers were determined as those with the predicted activity value $> \pm 3.0$ standardized residuals. The leverage value (h) measures the distance from the centroid of the modeled space. A warning leverage (critical hat value, h*) [30] was used to identify structural/MOA influential compounds ($h > h^*$ denoting high-leverage chemicals). In a Williams plot [30], the leverage values were mapped against the standardized residuals to define the structural/MOA and the response spaces visually. Finally, the AD was reported as the percentile coverage for the training ($AD_{Train}$) and test ($AD_{Test}$) set, respectively. Moreover, the Insubria graph [15] of leverage values against calculated/predicted activity values was used to visualize the interpolated ($h < h^*$ denoting chemicals inside the structural/MOA AD of the training set) and extrapolated ($h > h^*$ characterizing chemicals outside the structural/MOA AD) predictions for all the datasets considered in this study. In this case, the response AD was the prediction range of the model.

The standarization approach [31] is based on the assumption that in case of normal distribution, 99.7% of the population will remain within the range mean $\pm 3.0$ standard deviation (SD). Thus, all molecular descriptors are first standardized. Afterwards, any compound outside this zone is dissimilar to the rest and majority of the compounds. Thus, if the standardized value for descriptor *i* of compound *k* is more than 3, then the compound should be an X-outlier (if in the training set) or outside AD (if in the test set) based on descriptor *i*.

In the distance based methods [32–34], the distance between the chemical and the center of the training data set is computed. The threshold, for both Euclidean and City block distances, is the largest distance between the training set data points and the center of the training data set. Furthermore, the distance between the test samples and the center of the dataset is computed. The test points with a distance greater than the computed threshold are considered outliers. The AD was reported as the percentile coverage of the test set ($AD_{Test}$).

In the k-nearest neighbours method [34], the distance between every train compound and its k-nearest neighbours in the training set is computed. A threshold is calculated as the largest of these distances. Subsequently, the distance between every test compounds and its k-nearest neighbours in the training set is computed. If the calculated distance values of test set compounds is within the defined threshold, then the prediction of these compounds are considered to be reliable. In this method the k value was set to 3. The AD was reported as the percentile coverage of the test set ($AD_{Test}$).

The final consensus value on the training compounds is computed as a mean of the leverage and standardization methods, while the consensus on the test set is computed as the mean of all the different approaches.

### Selection of the final model

Among the generated candidate models, the one with the best compromise between statistical robustness, predictive performance, widest AD, and smallest dimension was selected as the final model. To this end, all 81 alternative specifications were filtered based on multiple up-to-date statistical acceptance criteria (highlighted in Additional file 1). Only the models both satisfying the internal and external validation requirements, and providing 100% $AD_{Test}$ coverage, with the consensus method, were considered eligible. Moreover, the transformation parameters ($\alpha ^*$,$\gamma ^*$) achieving the best predictive performance were selected as the set of indices of eligible solutions by solving $(\alpha ^*\gamma ^*) = arg min_t ( E(\alpha _t,\gamma _t);$ $t \in I)$ with $I \subseteq \left\{ 1, \ldots ,81 \right\}$. Finally, the model satisfying all eligibility criteria, consisting of the smallest number of structural/MOA features, and with the widest $AD_{Train}$ coverage was selected as the ultimate model.

### Application of the final model

The optimal model was applied to a set of external compounds for which the $logK_{HSA}$ is unavailable. To this purpose, an independent set of 799 drugs from the CMap dataset [16] with gene expression data available on both MCF7 and PC3 cell lines was considered. SDF files for these compounds were retrieved from PubChem [13] and fed into DRAGON v. 7.0 [14] to generate molecular descriptors. Gene expression data was preprocessed similarly to the dataset of 59 compounds, as described above. The list of drugs in the external dataset is available in Additional file 1. The TSNE projection technique [35] was used to visualize the distribution of the albumin and the external datasets based on the six MDs/MOA features of the QSMARt model as well as the three MOA features and three MDs.

## Results and discussion

### QSMARt predictive model for the binding affinity to HSA

Here, we built an integrated model (QSMARt) comprising molecular descriptors and MOA features to predict binding affinity to human serum albumin. To this end, we derived 81 candidate models by applying a Lasso penalty parameter optimisation. The 81 models and their evaluation metrics are reported in Additional file 1. The full lists of selected MDs and genes, along with their occurrence frequencies, are available in Additional file 1. Upon rigorous evaluation based on the OECD validation principles [2], we selected a final model of six structural/MOA features: three molecular descriptors and three gene expression patterns (Eq. 3).

3 $$\begin{aligned} LogK_{HSA}&= -0.372 + 0.012 |Mor23i|^{1.25} - 0.042 |N-072|^{1.25}\\&\quad+ 0.139 |ALOGP|^{1.25} -2.980 |MCF7\_ENSG00000112115|^{1.75}\\&\quad-0.075|PC3\_ENSG00000197646|^{1.75} -0.216|PC3\_ENSG00000276644|^{1.75} \end{aligned}$$

**Fig. 1:**
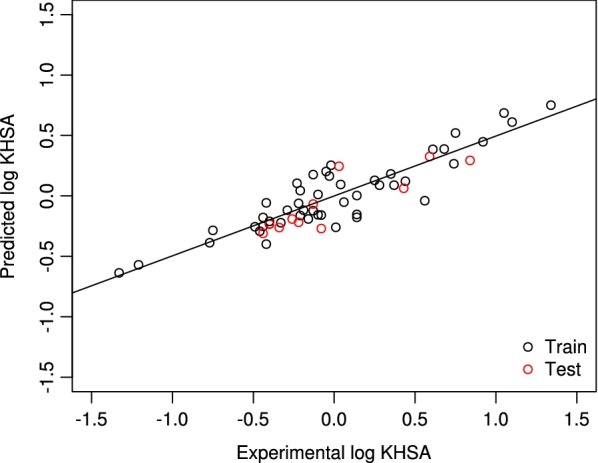
Predicted $logK_{HSA}$ versus experimental $logK_{HSA}$ values of training set (black) and test set (red) chemicals

**Fig. 2:**
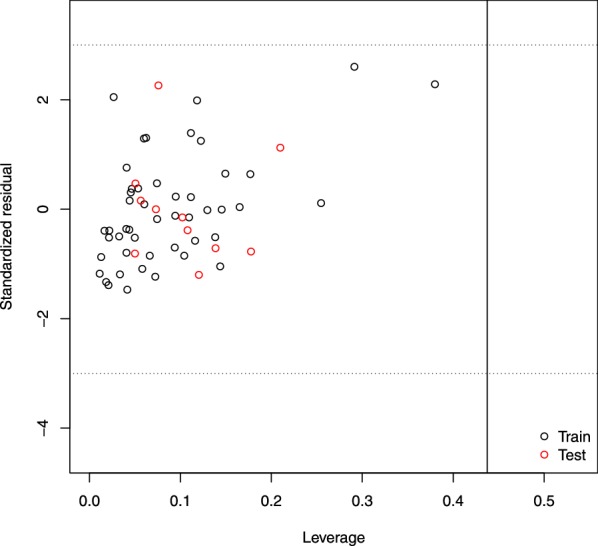
Standardized residuals versus leverage values of training set (black) and test set (red) chemicals (Williams plot). Dashed lines indicate $3.0\sigma$ interval. Vertical line set at the warning leverage (critical hat value, $h^* = 0.438$)

A good concordance between the predicted and experimental data is shown in Fig. 1. Our model fulfils the criteria regarding the goodness of fit and the internal and external validation requirements, as shown in Additional file 1. Moreover, the final hybrid model statistics passed all the recommended thresholds except the CCC metric (Additional file 1). Indeed, the $Q_{L10-Out}$, $R^2_{tr}$, $R^2_{te}$, $Q^2_{F_1}$, $Q^2_{F_2}$, $Q^2_{F_3}$ are greater than 0.6, but, the $CCC_{Te}$ value is smaller than 0.85. Next, we defined the AD of our model based on the consensus strategy. Noteworthily, all chemicals of the test set are inside the AD spaces (Additional file 1), suggesting that all the predictions were reliably interpolated. For visualization purposes we show the AD computed by means of the the leverage approach [30] in Fig. 2.

### Impact of the integration approach and comparison to sub-models

Next, in order to evaluate the impact of the integration strategy, we compared our QSMARt integrated final model with the two obtained by applying our approach to the MDs and genes separately. We ran the SVA methodology for the same nine $\alpha$ and $\gamma$ values and we obtained 9 models for the MDs, while only two models were obtained by using the genes alone, since no fitting was obtained for 7 $\gamma$ values. The best models for the MDs and genes respectively are the following:

4 $$\begin{aligned} LogK_{HSA}&= -0.335 + 0.077 |Mor23i|^{0.11} - 0.012 |R8s.|^{0.11} + 0.007 |C.040|^{0.11} \\&\quad- 0.061 |N-072|^{0.11} + 0.062 |ALOGP|^{0.11} - 0.003 |CATS3D_06_AP .|^{0.11} \\&\quad+ 0.0006 |piPC08|^{0.11} - 0.010 |GATS2i|^{0.11} + 0.001 |SpMax1_Bh.v.|^{0.11} \end{aligned}$$

5 $$\begin{aligned} LogK_{HSA}&= {} 0.042 + 3.84 |MCF7\_ENSG00000185950|^{0.16} \\&\quad-11.163 |MCF7\_ENSG00000112115|^{0.16} -0.758 |MCF7\_ENSG00000135100|^{0.16} \\&\quad+0.193 |PC3\_ENSG00000128228|^{0.16} + 0.0007 |PC3\_ENSG00000168209|^{0.16}\\&\quad+ 0.040|PC3\_ENSG00000110619|^{0.16} - 0.755|PC3\_ENSG00000064687 |^{0.16} \\&\quad-1.310|PC3\_ENSG00000168875|^{0.16} -9.301|PC3\_ENSG00000276644 |^{0.16} \end{aligned}$$

As evidenced in Additional file 1, the QSMARt model is characterized by overall better values of all the relevant diagnostic statistics. This analysis, hence, highlighted an overall better statistical performance of the integrated QSMARt model (Eq. 3) over the two competitor models (Eq. 4 and Eq. 5). In particular, the QSMARt model consists of less features, since it uses only 3 MDs and 3 genes, while the other two models use 9 MDs and 9 genes, respectively. Furthermore, the model coming from the genes does not show any predictive capability on the test set ($R^2_{test} = 0.10$) although its $R^2_{train} = 0.61$. On the other hand the model obtained by using only MDs has good predictive capabilities, even thought they are smaller than the one obtained by the QSMARt model. Furthermore, when comparing the distributions of the $Q^2$, $Q^2F_1$,$Q^2F_2$, $Q^2F_3$ and *CCC* metrics that are computed with the RSVA method, the performances of the QSMARt model are better than those of the other two models (Additional file 5: Fig. S2).

### Mechanistic interpretation of the features included in the QSMARt model

Mechanistic interpretation of the molecular descriptors included in a model is an OECD principle of QSAR validation [2]. The hybrid model was built with the following MDs: Mor23i, N-072, and ALOGP. Mor23i is a measure of the pair-wise interatomic distance and ionization potential [36]. Ionization potential is the amount of energy required to extract one electron from a chemical system, i.e., a measure of the capability of a molecule to give the corresponding cation. Mor23i and $logK_{HSA}$ are positively correlated (Eq. 3), implying that the higher the ionization potential, the higher the HSA binding affinity. This further suggests that electron-pair acceptors (Lewis-acids) have higher binding affinity to HSA. Given the positive coefficient of Mor23i in our model equation, compounds with more acidic properties have higher binding affinity to HSA. On the other hand, due to the mathematical background, the distance between two influential atoms may majorly define the descriptor [36]. Therefore, a more detailed interpretation could be useful for molecular design purposes.

N-072 is a descriptor counting the nitrogen-centered fragments of RCO-N< or > N-X=X in a chemical structure, where R is any group bound through carbon, X is any electronegative atom, such as oxygen, nitrogen, sulfur, phosphorus, and halogens, - is single and = is double bonds, respectively [3]. The negative coefficient in the final model indicates that chemicals with N-072 fragments show less affinity to HSA binding. Similarly, N-072 was reported elsewhere as affecting the relative fluorescence intensity ratio [37].

ALOGP is a measure of hydrophobicity as the logarithm of n-octanol/water partition coefficient. Based on the Ghose-Crippen method [38], it is calculated as the summation of atomic contributions to overall molecular hydrophobicity. Clearly, having a positive coefficient in the model equation, ALOGP explains an increased affinity to HSA binding. It has been already reported in relation to binding affinity to HSA [12, 39]. Furthermore, earlier studies on the crystallographic structure of HSA and binding affinity evidenced that the binding sites of HSA are mainly composed of hydrophobic residues, further revealing that hydrophobicity is a major property encoding the binding affinity, as reviewed in [40, 41].

Three genes are included in the final QSMARt model, namely Interleukin 17A (IL17A) from the MCF7, Programmed Cell Death 1 Ligand 2 (PD-L2), and Dachshund Family Transcription Factor 1 (DACH1) from the PC-3 transcriptomic datasets, respectively. The expression of IL17A is documented in the MCF7 cell line, where it has been tested as target of chemotherapeutic strategies aiming at altering autophagic ability of breast cancer cell lines [42]. Alteration of the expression of PD-L2, a ligand of PD-1, has been observed both in prostate cancer in response to anti-PD-1 therapy [43]. DACH1 is a transcription factor expressed in prostate cancer, where its low expression is associated with higher malignant potential [44]. Interestingly, all these three genes have known immunomodulatory properties, either as pro-inflammatory (IL17A) or immunosuppressive (PD-L2 and DACH1). Since their QSMARt model coefficients are negative, the impact of drugs to alter their expression is inversely proportional to HSA binding affinity. These results, for instance, suggest that the serum supplementation in the cell culture medium and the compound dosages should be mutually adjusted when testing drugs in vitro, such as in the CMap experiments.

**Fig. 3:**
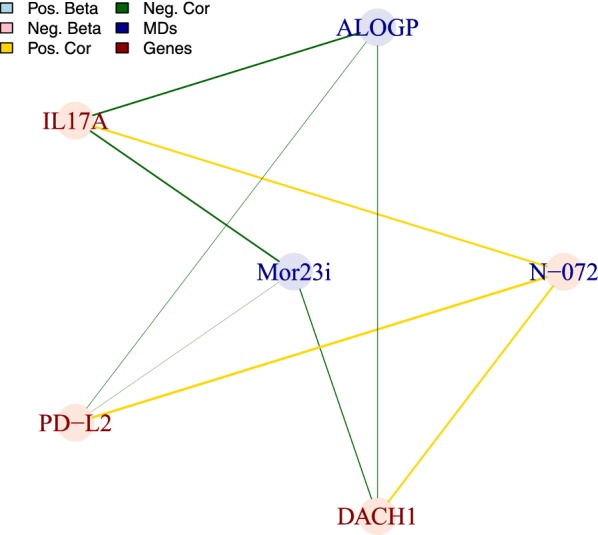
Correlation graph of the six MDs/MOA features of the QSMARt model. Vertex color represent the sign of the associated beta value while edge colors show the sign of the correlation of the features across the X dataset

Next, we considered the correlation between the three gene expression patterns and the three MDs included in the QSMARt model (Fig. 3). All the genes in the model were negatively correlated with Mor23i and ALOGP, and positively correlated with N-072, respectively. These results imply that potentially less acidic (lower values of Mor23i) and less lipophilic compounds (lower values of ALOGP) have a higher impact in altering the expression of these three genes.

Altogether, according to the QSMARt model, compounds with higher values of ionization potential and hydrophobicity, and less nitrogen-centered residuals, as well as lower expression alteration of the immunomodulatory genes IL17A, PD-L2 and DACH1, have higher binding affinity to HSA.

Taken together, these results provide an extended mechanistic interpretation of the interactions of chemicals and biological systems by providing direct associations between specific structural and biological properties of the exposure.

### Application of the QSMARt model

**Fig. 4:**
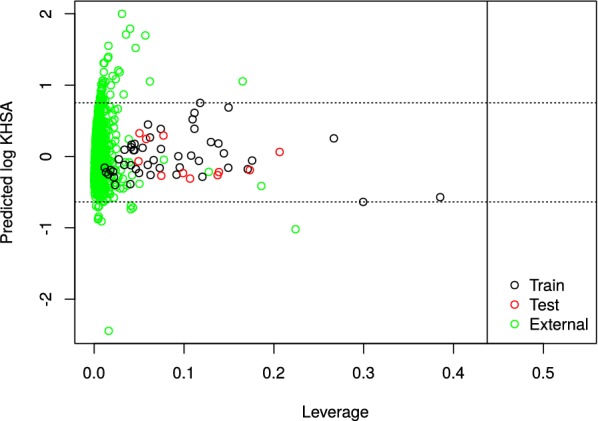
Predicted $logK_{HSA}$ by Eq. 3 versus leverage values of training set (black), test set (red), and external set (green) chemicals (Insubria graph). Dashed lines indicate the model prediction range. Vertical line set at the warning leverage (critical hat value, $h^* = 0.438$)

**Fig. 5:**
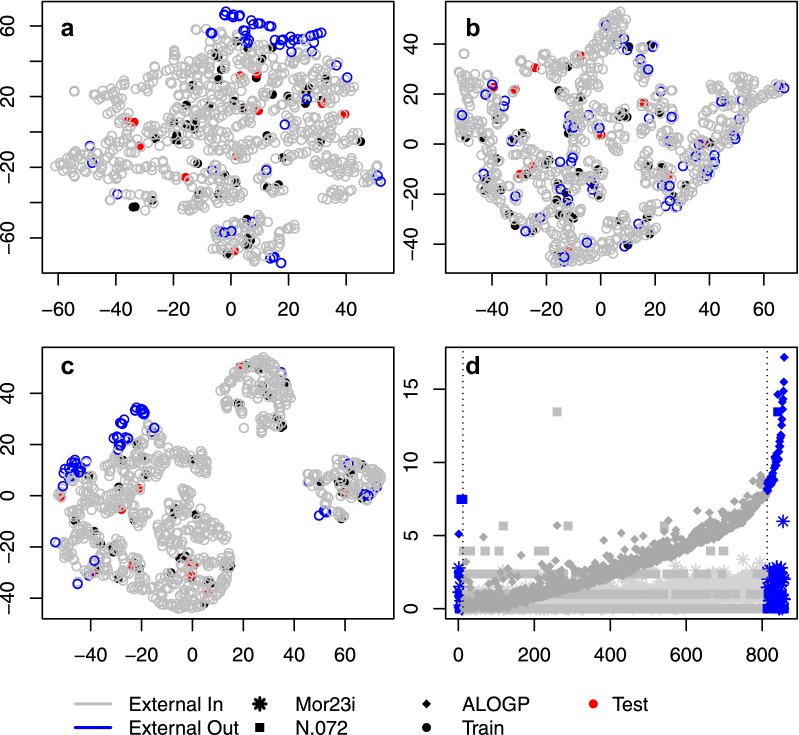
TSNE projection of the drugs in the albumin and external dataset. The projection was performed by using the set of genes and MDs (**a**), only the genes (**b**) and only the MDs (**c**) in the optimal hybrid model. The outliers are in the border area of the dataset for the molecular descriptors (**c**), while they are similar to the rest of the external set fort the gene log-fold change (**b**). Likewise, the outliers still appear on the border for the combined two sets of features (**a**). In panel (**d**) the values of the three MDs is plotted (y axis) for the drugs in the albumin and external dataset (x axis). The drugs are ordered based on their predicted $logK_{HSA}$ value. Drugs from the external set that falls in the model prediction range are marked in gray, while the ones that are outside the range are marked in blue. Drugs in the training set are marked in black while drugs in the test set are marked in red

Finally, we tested the performance of our QSMARt model in predicting the $logK_{HSA}$ for an independent set of 799 compounds extracted from the CMap dataset. With 741 chemicals in the AD, our model provided a remarkable prediction coverage of 93% (Fig. 4). It is noteworthy to emphasize that no external chemicals falling outside the structural/MOA feature domain were identified. However, 58 drugs appeared outside the model prediction range and were further investigated. For this, we inspected the distribution of the different subsets of compounds in a projected space based on the six MDs/MOA features of the QSMARt model (Fig. 5a) as well as the three MOA features (Fig. 5b) and three MDs (Fig. 5c) considered separately. This analysis evidenced that the external set chemicals falling outside the model prediction range show less structural commonalities with the rest of the compounds (Fig. 5c) but are genomically confounded with the others (Fig. 5b). Thus, we further investigated the value of the MDs for the external dataset and, as we can see from Fig. 5d, drugs falling outside the prediction range of our model have higher value for the ALOGP MD.

### Biological relevance of the QSMARt model

In order to better understand the possible impact of the QSMARt model, we investigated its performance on drugs grouped by the ATC (Anatomical, Therapeutic, Chemical) code system as defined by the World Health Organization (WHO) [45]. The ATC codes classify the drugs into different groups in accordance with the organ or system on which they act and their chemical, pharmacological, and therapeutic properties. We performed our analyses by considering the anatomical subgroup (level 1) and the therapeutic subgroup (level 2) of the ATC codes. We investigated the relationship between the experimental *vs.* predicted logKHSA values, of the 59 drugs present in our dataset, and their grouping in ATC level 1 and 2 (Additional file 6: Fig. S3). This analysis highlights that the two drugs cefuroxime and amoxicillin, belonging to the ATC class J (any-inflammatory), show the lowest range of experimental and predicted logKHSA. Likewise, a large group of ATC class C compounds (cardiovascular system) are in the mid range of the distribution, while four ATC class N (nervous system) are grouped in the highest range of the experimental/predicted logKHSA. Next, we inspected the larger set of 799 drugs used for the external validation, for which no experimental value of logKHSA was available. In this case, we looked at the distribution of the predicted logKHSA values in the level 1 and level 2 ATC codes (Additional files 7: Fig. S4 and 8: Fig. S5). Also, this analysis shows that the compounds belonging to the ATC class J (anti-inflammatory) have the lowest levels of predicted logKHSA. On the opposite, drugs of the ATC class A (digestive system), G (genitourinary system) and N (nervous system) have the highest predicted logKHSA. These results confirm our observations on the 59 drugs present in our discovery set.

The genes selected in our model are involved in several signalling pathways, especially in cancer and immune signalling. Thus, we investigated their expression values between immunomodulatory and non immunomodulatory compounds. We identified the level 2 classes L03 and L04 to be immunostimulant and immunosuppressant, respectively. Unfortunatelly, none of the compounds available in the Connectivity map data set belong to the class L03, while four are annotated as L04. To perform the comparison, we selected the compound structurally least similar to each of the L04 drugs in the Connectivity Map dataset, and plotted the respective expression values for each of the three genes included in our final model (Additional file 9: Fig. S6). While MCF7_ENSG00000112115 And PC3_ENSG00000197646 did not show any difference, the gene PC3_ENSG00000276644 showed a trend with higher expression in L04 drugs as compared to their least similar ones.

## Conclusion

In this study, we proposed a computational strategy to define quantitative models of structural and mechanism of action-activity relationships (QSMARt). Moreover, we investigated the effectiveness of hybrid QSMARt model comprising both MDs and MOA information to better explain the biological mechanisms underlying endpoints of interest. We applied our methodology to predict human serum albumin (HSA) binding, obtaining a statistically robust and validated model that provides new venues for the interpretation of the chemical-biological interactions. QSMARt models are promising complementary tools to develop new safe- and useful-by-design compounds.

## Additional file

**Additional file 1.** This file contains the following supplementary tables:** Table S1:** Dataset;** Table S2:** Parameters and criteria considered for goodness of fit, internal and external validation;** Table S3:** External dataset with predicted *LogK*_*hsa*_; **Table S4:** Molecular descriptors appearing in the models; **Table S5:** Genes appearing in the models; **Table S6:** Summary of the models parameters and evaluation metrics.

**Additional file 2.** This file contains the formal descriptions of the methodology and the RSVA algorithm

**Additional file 3.** File containing the R functions and scripts to create the integrative model.

**Additional file 4.** **Fig. S1**. Estimation of the optimal λ; value with the RSVA algorithm.

**Additional file 5.** **Fig. S2**. Comparison of the model validation curves.

**Additional file 6.** **Fig. S3**. Scatterplot of experimental vs. predicted logK_HSA_ values of the 59 drugs, coloured by ATC codes level 1 and 2.

**Additional file 7.** **Fig. S4**. Boxplot of the predicted logK_HSA_ values of the 799 external compounds coming from CMap dataset grouped by ATC codes level 1.

**Additional file 8.** **Fig. S5**. Boxplot of the predicted logK_HSA_ values of the 799 external compounds coming from CMap dataset grouped by ATC codes level 2.

**Additional file 9.** **Fig. S6**. Boxplot of the expression values of the three selected genes, grouped by immunosuppressant and their less similar drugs.

## Data Availability

The datasets supporting the conclusions of this article are included within the article as Additional Files.

## Abbreviations

AD: applicability domain
CMap: connectivity map
HL60: leukemia cell line
HSA: human serum albumin
Lasso: least absolute shrinkage and selection operator
MCF7: breast cancer cell line
MDs: molecular descriptors
MOA: mechanism of action
OECD: organization for economic co-operation and development
PC3: prostate cancer cell line
QSAR: quantitative structure-activity relationship
QSMARt: quantitative structure and mechanism of action-activity relationship
