# Supplementary material for: An integrated quantitative structure and mechanism of action-activity relationship model of human serum albumin binding
Source: J Cheminform. 2019 Jun 6;11:38. doi: 10.1186/s13321-019-0359-2 (PMC6551915; doi:10.1186/s13321-019-0359-2)
Supplement: Supplementary file 2 — Additional file 2. This file contains the formal descriptions of the methodology and the RSVA algorithm [file 13321_2019_359_MOESM2_ESM.pdf]

An integrated quantitative structure and mechanism of action-activity relationship model of human serum albumin binding

Angela Serra[1], Serli Önlü[1], Pietro Coretto[2], and Dario Greco[1,3,*]

[1]Faculty of Medicine and Health Technology, Tampere University, Tampere, Finland.
[4]DISES, STATLAB, University of Salerno, Fisciano, Italy
[3]Institute of Biotechnology, University of Helsinki, Finland
[*] *To whom the correspondence should be addressed: dario.greco@staff.uta.fi*

Feature selection and data transformation

After the preprocessing of molecular descriptors and gene expression data, three data matrices are obtained:

- $\boldsymbol{A} = (a_{ij})$ is the molecular descriptors (MD) data matrix, this is of dimension (59×1196). A column vector of $\boldsymbol{A}$ corresponds to a MD measured across the 59 drugs;
- $\boldsymbol{B} = (b_{ij})$ is the matrix of log-foldchange for the genes coming from the MCF7 cell line ($\mathrm{MCF7_{FC}}$). This matrix is of dimension (59×11868), and each column of $\boldsymbol{B}$ represents log-foldchange for a certain gene over the 59 drugs;
- $\boldsymbol{C} = (c_{ij})$ is the matrix of log-foldchange for the genes coming from the PC3 cell line ($\mathrm{PC3_{FC}}$). This has the same dimension of $\boldsymbol{B}$, also the measurements are organized similarly.

Let $\boldsymbol{X}$ be the matrix obtained by binding the matrices $\boldsymbol{A}, \boldsymbol{B}$ and $\boldsymbol{C}$ by columns. The full data matrix $\boldsymbol{X}$ is now of dimension (59×24932), which means that it contains measurements of $p = 24932$ features over $n = 59$ drugs. Let $\boldsymbol{y}$ be the vector of the $\log \mathrm{K_{HSA}}$ values measured on the 59 drugs. We want to construct a model that relates the $\boldsymbol{y}$ and the features in $\boldsymbol{X}$ with good predictive performance. The usual driving assumption is that the relationship between the expectation of $\boldsymbol{y}$ and the features is sparse, meaning that only a small subset of the p features is relevant to predict $\boldsymbol{y}$.

Existing literature approached the problem by estimating a linear model where $\boldsymbol{y}$ is regressed against MD descriptors, and relevant MD descriptors have been found by using the LASSO method of [13] optimized for predictive performance using the k-fold cross-validation. In this paper we address the issue whether genomic information lead to improved predictive performance, therefore we look for a reasonably small number of MD

and genes that would jointly better predict the outcome $\boldsymbol{y}$. In other words, one could assume that the data generating process is well represented by the usual linear model

$$\boldsymbol{y} = \boldsymbol{X}\boldsymbol{\beta} + \text{stochastic error}, \tag{1}$$

where $\boldsymbol{\beta}$ is a sufficiently sparse vector of coefficients of dimension equal to the number of features. The LASSO method combined with cross-validation can be used to estimate the non-zero components of $\boldsymbol{\beta}$ corresponding to the relevant subset of features.

Exploratory analysis on the datasets considered in this paper has shown that certain power transformations of the original features in $\boldsymbol{X}$ lead to a better association with the outcome variable $\boldsymbol{y}$. Fix $\alpha > 0$ and consider the matrix $\boldsymbol{A}(\alpha) = (|a_{ij}|^{\alpha})$, that is $\boldsymbol{A}(\alpha)$ is obtained by taking the absolute value of its elements raised to the power α. Fix $\gamma > 0$ and take $\boldsymbol{B}(\gamma) = (|b_{ij}|^{\gamma})$ and $\boldsymbol{C}(\gamma) = (|c_{ij}|^{\gamma})$. Exploratory analysis has shown that for these transformed features weak correlations with the $\boldsymbol{y}$ are somewhat downgraded, while strong correlations are emphasized. Therefore the issue whether transformed features have better predictive power is addressed. Let $\boldsymbol{X}(\alpha, \gamma)$ be the (59×24932) matrix obtained by binding the matrices $\boldsymbol{A}(\alpha), \boldsymbol{B}(\gamma)$ and $\boldsymbol{C}(\gamma)$ by columns. We now consider the following model

$$\boldsymbol{y} = \boldsymbol{X}(\alpha, \gamma)\boldsymbol{\beta} + \text{stochastic error}. \tag{2}$$

First, note that the same power transformation has been used for the two groups of genomic features in matrices $\boldsymbol{B}$ and $\boldsymbol{C}$. This is because we assume two similar, but distinct, mechanisms driving the linear association between MD features and genomic features with respect to the outcome variable. Second, note that model (2) is nonlinear if we consider α and γ as structural parameters explaining in-sample biological relationships. In this case one could estimate α, γ, and $\boldsymbol{\beta}$ by penalized nonlinear least squares with a LASSO-type penalty achieving a sparse estimate of $\boldsymbol{\beta}$. Not only this is computationally challenging, but it also means that we attach to α and γ a structural/biological meaning that is hard to justify. On the other hand, for fixed α and γ the model (2) becomes a linear model as (1). Considering α and γ fixed, with only $\boldsymbol{\beta}$ being a structural/biological parameter to be estimated, is conceptually the same as replacing the original sample measurements $\boldsymbol{X}$ with a different version $\boldsymbol{X}(\alpha, \gamma)$ and looking for a linear model as in (1). Therefore, given pair a (α, γ), and $\lambda > 0$, the LASSO estimator of $\boldsymbol{\beta}$ is given by

$$\hat{\boldsymbol{\beta}} = \arg\min_{\boldsymbol{\beta}} \|\boldsymbol{y} - \boldsymbol{X}(\alpha, \gamma)\boldsymbol{\beta}\|_2^2 + \lambda \|\boldsymbol{\beta}\|_1, \tag{3}$$

where $\|\cdot\|_2$ is the euclidean norm, $\|\cdot\|_1$ is the ℓ^1 norm, λ is the LASSO penalty. For larger choice of λ, the term $\lambda \|\boldsymbol{\beta}\|_1$ in (3) penalizes the least square cost function at $\boldsymbol{\beta}$ vectors with too many too small βs coefficients. Depending on λ, the LASSO sets small regression coefficients exactly to zero, and nonzero components of $\hat{\boldsymbol{\beta}}$ correspond to the relevant features that are selected by the method.

The tripled $(\alpha, \gamma, \lambda)$ can be considered as methods' parameters tuned to achieve the best predictive power of the linear model (2) where only the $\boldsymbol{\beta}$ pretends to explain the structural/biological relationships between the transformed features and the outcome $\boldsymbol{y}$.

Random split validation is used to find the best combination of $(\alpha, \gamma, \lambda)$. The final assessment is made on test sample not used in any previous estimation or optimization task.

Let n be the sample size (number of rows of $\boldsymbol{X}$). Let $\dot{\boldsymbol{X}}$ and $\dot{\boldsymbol{y}}$ contain $n_0 = [80\%n]$ of the original samples, and let $\ddot{\boldsymbol{X}}$ and $\ddot{\boldsymbol{y}}$ contain the remaining $(n - n_0)$ samples used for the final assessment. Let $\{\lambda_m,\ m = 1, 2, \ldots, M\}$ a fixed grid of values for the LASSO penalty parameter. Fix the size of the training dataset $n_1 = [90\%n_0]$, and the size of the test dataset $n_2 = n_0 - n_1$. Random cross-validation is performed for a given pair (α, γ) as follows.

Random Split Validation Algorithm (RSVA)

Inputs: $\dot{\boldsymbol{X}}$,$\dot{\boldsymbol{y}}$, α, γ, $\{\lambda_m,\ m = 1, 2, \ldots, M\}$

1. **For** $k = 1, 2, \ldots, K$; **do**

   (1.a) Randomly split the dataset (without replacement) in a training dataset $(\dot{\boldsymbol{y}}_1, \dot{\boldsymbol{X}}_1)$ of size n_1, and a test set $(\dot{\boldsymbol{y}}_2, \dot{\boldsymbol{X}}_2)$ of size n_2

   (1.b) **For** $m = 1, 2, \ldots, M$; **do**

   (1.b.i) compute the LASSO solution

   $$\hat{\boldsymbol{\beta}}^{(k,m)} \leftarrow \arg\min_{\boldsymbol{\beta}} \left\| \dot{\boldsymbol{y}}_1 - \dot{\boldsymbol{X}}_1(\alpha, \gamma)\boldsymbol{\beta} \right\|_2^2 + \lambda_m \|\boldsymbol{\beta}\|_1$$

   (1.b.ii) compute the vector of the predicted values on the test sample:

   $$\hat{\boldsymbol{y}}_2 \leftarrow \dot{\boldsymbol{X}}_2(\alpha, \gamma)\hat{\boldsymbol{\beta}}^{(k,m)}$$

   (1.b.iii) compute the mean squared prediction error (MSPE):

   $$\mathrm{E}_{k,m} \leftarrow \frac{1}{n_1} \|\hat{\boldsymbol{y}}_2 - \dot{\boldsymbol{y}}_2\|_2^2$$

2. For all $m = 1, 2, \ldots, M$ compute the averaged MSPE across the random splits, and their estimated standard error

   $$\overline{\mathrm{E}}_m \leftarrow \frac{1}{K} \sum_{i=1}^{K} \mathrm{E}_{k,m}$$

   $$\mathrm{se}(\overline{\mathrm{E}}_m) \leftarrow \sqrt{\frac{1}{K(K-1)} \sum_{i=1}^{K} (\mathrm{E}_{k,m} - \overline{\mathrm{E}}_m)^2}$$

3. λ selection: let $m_o = \arg\min_m \{\overline{\mathrm{E}}_m,\ m = 1, \ldots, M\}$, and take

   $$\lambda^* = \max\{\lambda_m :\ \overline{\mathrm{E}}_o - 1.96\,\mathrm{se}(\overline{\mathrm{E}}_o) \le \overline{\mathrm{E}}_m \le \overline{\mathrm{E}}_o + 1.96\,\mathrm{se}(\overline{\mathrm{E}}_o)\}$$

   Let $\boldsymbol{\beta}(\alpha, \gamma)$ be the LASSO solution computed at $\lambda = \lambda^*$, and let $\overline{\mathrm{E}}(\alpha, \gamma)$ the corresponding average MSPE.

Output: $\boldsymbol{\beta}(\alpha, \gamma)$ and $\overline{\mathrm{E}}(\alpha, \gamma)$

A grid of 9 distinct α and γ values are considered, with $\alpha, \gamma \in \{0.1, 0.25, 0.5, 0.75, 1, 1.25, 1.5, 1.75, 2\}$. The random split validation algorithm is performed for all of the 81 distinct pairs (α_t, γ_t) for $t = 1, 2, \ldots, 81$.

For each of the 81 combinations, the relevant set of features $f_t = \beta(\alpha_t, \gamma_t)$ (for $t = 1, 2, \ldots, 81$) associated to the non null coefficients is identified. The samples in $(\dot{\boldsymbol{X}}, \dot{\boldsymbol{y}})$ are used to train a model with this set of feature and its associated α and γ parameter. After that, the model is used to predict the outcome $\log \mathrm{K_{HSA}}$ on the final test set $(\ddot{\boldsymbol{X}}, \ddot{\boldsymbol{y}})$. For these models the up-to-date internal and external validation metrics [7] are evaluated by applying a further random split validation strategy on the training dataset $(\dot{\boldsymbol{X}}, \dot{\boldsymbol{y}})$. The internal evaluation metrices are the following: coefficient of determination r^2_{Train}, mean squared error MSE_{Train} and MSE, leave-more-out correlation coefficient Q^2_{LMO}, new coefficient of determination following the Y-scambling procedure [10] $r^2_{Y_{src}}$, $Q^2_{Y_{src}}$. The Y-scrambling procedure, identifies a possible chance correlation between the toxicity and descriptors. In the Y-scrambling method, the toxicity variables of the training set randomly shuffled 100 times and new coefficient of determination $r^2_{Y_{src_i}}$ and $Q^2_{Y_{src_i}}$ are evaluated. $r^2_{Y_{src}}$, $Q^2_{Y_{src}}$ are the mean of the 100 shuffled values. Low values of these parameter indicate that the original model was not built by chance correlation. The external evaluation metrices are the following: coefficient of determination r^2_{Test}, mean squared error MSE_{Test}, Q^2_{F1} [12], Q^2_{F2} [11], Q^2_{F3} [3], concordance correlation coefficient CCC_{Test}[1, 2], r^2_m. Along with these metrics the applicability domain, based on the leverage method, is also computed both for the training AD_{Train} and the test AD_{Test} datasets. The measure referred as $_{Train}$ are computed only once on the prediction of the model fitted on the training dataset $(\dot{\boldsymbol{X}}, \dot{\boldsymbol{y}})$. The measure referred as $_{Test}$ are computed only once on the predicted value of the test dataset $(\ddot{\boldsymbol{X}}, \ddot{\boldsymbol{y}})$. All the others are computed on 100 random split of the dataset $(\dot{\boldsymbol{X}}, \dot{\boldsymbol{y}})$ in a training and validation set and are computed as the mean value of the metric computed on the internal validation dataset. The 81 models are then filtered based on threshold already estrablished in literature: $r^2 > 0.6$, $Q^2 > 0.5$, $r^2_{TEST} > 0.6$ [6], Q^2_{F1},Q^2_{F2} and $Q^2_{F3} > 0.6$, $CCC_{Test} > 0.85$ [1], $r^2_m > 0.5$ [9], $AD_{test} = 100$. Only the solutions that satisfy these requirements are considered eligible. Finally the transformation parameters (α^*, γ^*) achieving the best preditive performance with le less number of feature, is selected as $(\alpha^*, \gamma^*) = \arg\min_t \{\overline{\mathrm{E}}(\alpha_t, \gamma_t);\ t \in I\}$ with $I \subseteq \{1, \ldots, 81\}$ the set of indices of eligible solutions.

The LASSO solution in step (1.b.i) of RSVA is computed using the coordinate descendent algorithm implemented in the `glmnet` R package of [5]. Although k-fold cross-validation is a much more popular choice for managing the bias-variance tradeoff [14, 4], in our experiments, the random splitting strategy above with $K = 100$ gave a much more stable performance. In particular the increase in the number of splittings allowed a better estimate of the $\mathrm{se}(\overline{\mathrm{E}}_m)$ in Step 2 of the RSVA, and this is functional to the $\lambda-$selection strategy of Step 3. The optimal λ is typically chosen to minimize the estimated expected MSPE, this would correspond to the LASSO solution computed at $\lambda = \lambda_{m_o}$. A larger value of lambda implies a larger penalty, and therefore a sparser solution implying a smaller number of relevant feature selected. In Step 3 of the RSVA we select the largest λ value such that the corresponding averaged MSPE is still within the 95%-confidence interval (derived using the normal asymptotic approximation) around the solution achieving the the lowest overall averaged MSPE. Therefore, we select the sparsest solution that is statistically equivalent to the one achieving the lowest overall averaged MSPE. This strategy allows to select a more parsimonious model (less features) without compromising the predictive performance achieved on the final test set. This approach is also suggested in [8], although

they suggest a smaller confidence level.

References

[1] Nicola Chirico and Paola Gramatica. Real external predictivity of qsar models: how to evaluate it? comparison of different validation criteria and proposal of using the concordance correlation coefficient. *Journal of chemical information and modeling*, 51(9):2320–2335, 2011.

[2] Nicola Chirico and Paola Gramatica. Real external predictivity of qsar models. part 2. new intercomparable thresholds for different validation criteria and the need for scatter plot inspection. *Journal of Chemical Information and Modeling*, 52(8):2044–2058, 2012.

[3] Viviana Consonni, Davide Ballabio, and Roberto Todeschini. Comments on the definition of the q2 parameter for qsar validation. *Journal of chemical information and modeling*, 49(7):1669–1678, 2009.

[4] Bradley Efron and Trevor Hastie. *Computer age statistical inference*, volume 5 of *Institute of Mathematical Statistics (IMS) Monographs*. Cambridge University Press, New York, 2016.

[5] Jerome Friedman, Trevor Hastie, and Robert Tibshirani. Regularization paths for generalized linear models via coordinate descent. *Journal of Statistical Software*, 33(1):1–22, 2010.

[6] Alexander Golbraikh and Alexander Tropsha. Beware of q2! *Journal of molecular graphics and modelling*, 20(4):269–276, 2002.

[7] Paola Gramatica and Alessandro Sangion. A historical excursus on the statistical validation parameters for qsar models: a clarification concerning metrics and terminology. *Journal of chemical information and modeling*, 56(6):1127–1131, 2016.

[8] Trevor Hastie, Robert Tibshirani, and Jerome Friedman. *The Elements of Statistical Learning*, volume 1. Springer New York, 2009.

[9] Probir Kumar Ojha, Indrani Mitra, Rudra Narayan Das, and Kunal Roy. Further exploring rm2 metrics for validation of qspr models. *Chemometrics and Intelligent Laboratory Systems*, 107(1):194–205, 2011.

[10] Christoph Rücker, Gerta Rücker, and Markus Meringer. y-randomization and its variants in qspr/qsar. *Journal of chemical information and modeling*, 47(6):2345–2357, 2007.

[11] Gerrit Schuurmann, Ralf-Uwe Ebert, Jingwen Chen, Bin Wang, and Ralph Kuhne. External validation and prediction employing the predictive squared correlation coefficient-test set activity mean vs training set activity mean. *Journal of Chemical Information and Modeling*, 48(11):2140–2145, 2008.

[12] Leming M Shi, Hong Fang, Weida Tong, Jie Wu, Roger Perkins, Robert M Blair, William S Branham, Stacy L Dial, Carrie L Moland, and Daniel M Sheehan. Qsar models using a large diverse set of estrogens. *Journal of Chemical Information and Computer Sciences*, 41(1):186–195, 2001.

[13] Robert Tibshirani. Regression shrinkage and selection via the lasso. *Journal of the Royal Statistical Society. Series B. Methodological*, 58(1):267–288, 1996.

[14] Yongli Zhang and Yuhong Yang. Cross-validation for selecting a model selection procedure. *Journal of Econometrics*, 187(1):95–112, 2015.
