# Supplementary material for: An integrated quantitative structure and mechanism of action-activity relationship model of human serum albumin binding
Source: J Cheminform. 2019 Jun 6;11:38. doi: 10.1186/s13321-019-0359-2 (PMC6551915; doi:10.1186/s13321-019-0359-2)
Supplement: Supplementary file 4 — Additional file 4. Fig. S1. Estimation of the optimal λ; value with the RSVA algorithm. [file 13321_2019_359_MOESM4_ESM.pdf]

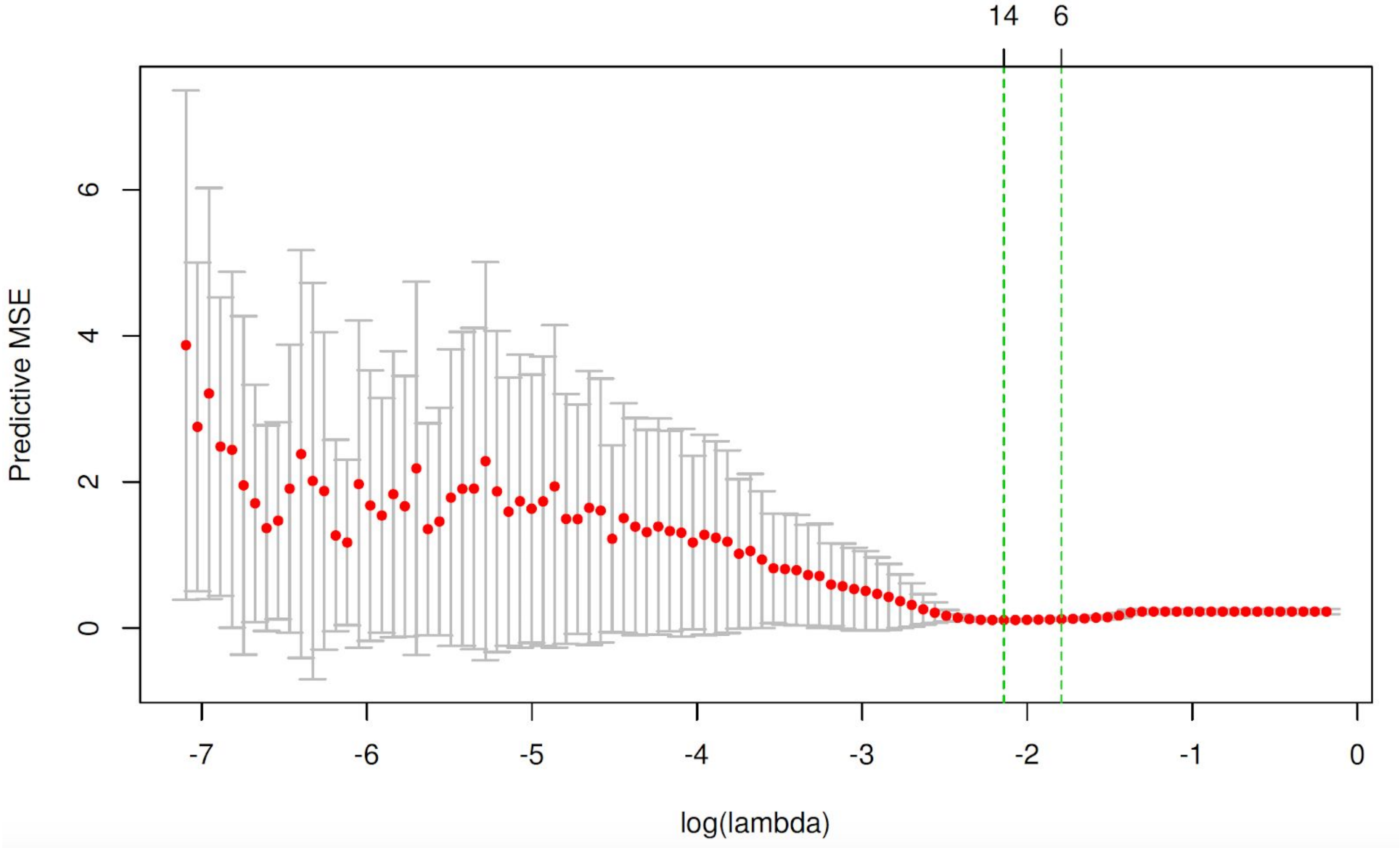

Figure S1: Estimated MSE for the 100 values. X-axis reports the logarithms of the values. Red dots show mean MSE values while vertical bars show their standard deviation. Optimal values are identified by the green dashed lines. The selected lambda value is 0.166. With this shrinking parameter, the hybrid model selected 6 features of which 3 are molecular descriptors and 3 are genes.
