# Supplementary material for: An integrated quantitative structure and mechanism of action-activity relationship model of human serum albumin binding
Source: J Cheminform. 2019 Jun 6;11:38. doi: 10.1186/s13321-019-0359-2 (PMC6551915; doi:10.1186/s13321-019-0359-2)
Supplement: Supplementary file 5 — Additional file 5. Fig. S2. Comparison of the model validation curves. [file 13321_2019_359_MOESM5_ESM.pdf]

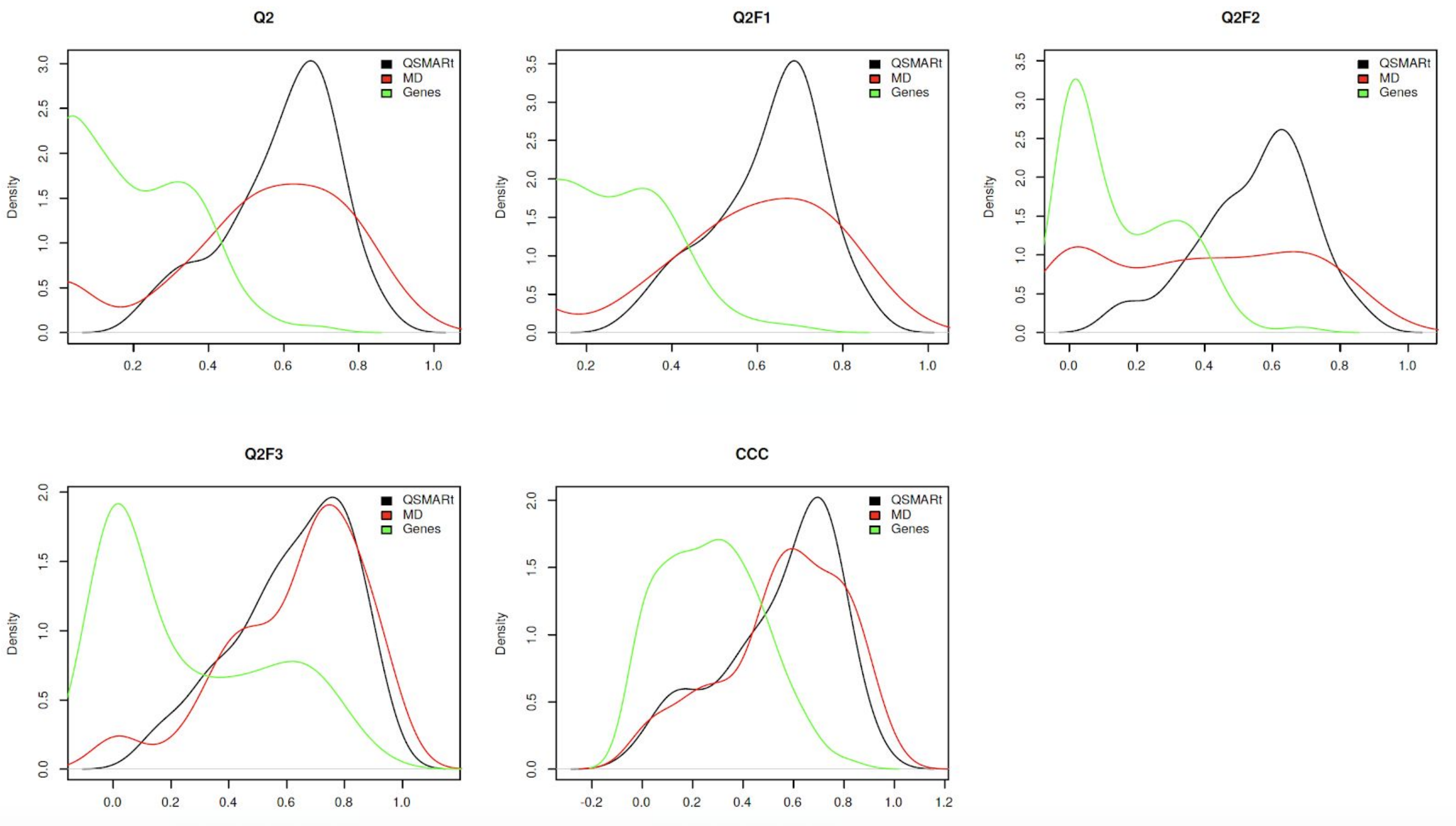

Figure S2: distributions of the Q^2, Q^2F_1, Q^2F_2, Q^2F_3 and CCC metrics computed by 100 repetitions of the RSVA method for the QSMARt model and the model obtained by using only the genes and MDs values.
