# Supplementary material for: An integrated quantitative structure and mechanism of action-activity relationship model of human serum albumin binding
Source: J Cheminform. 2019 Jun 6;11:38. doi: 10.1186/s13321-019-0359-2 (PMC6551915; doi:10.1186/s13321-019-0359-2)
Supplement: Supplementary file 6 — Additional file 6. Fig. S3. Scatterplot of experimental vs. predicted logKHSA values of the 59 drugs, coloured by ATC codes level 1 and 2. [file 13321_2019_359_MOESM6_ESM.pdf]

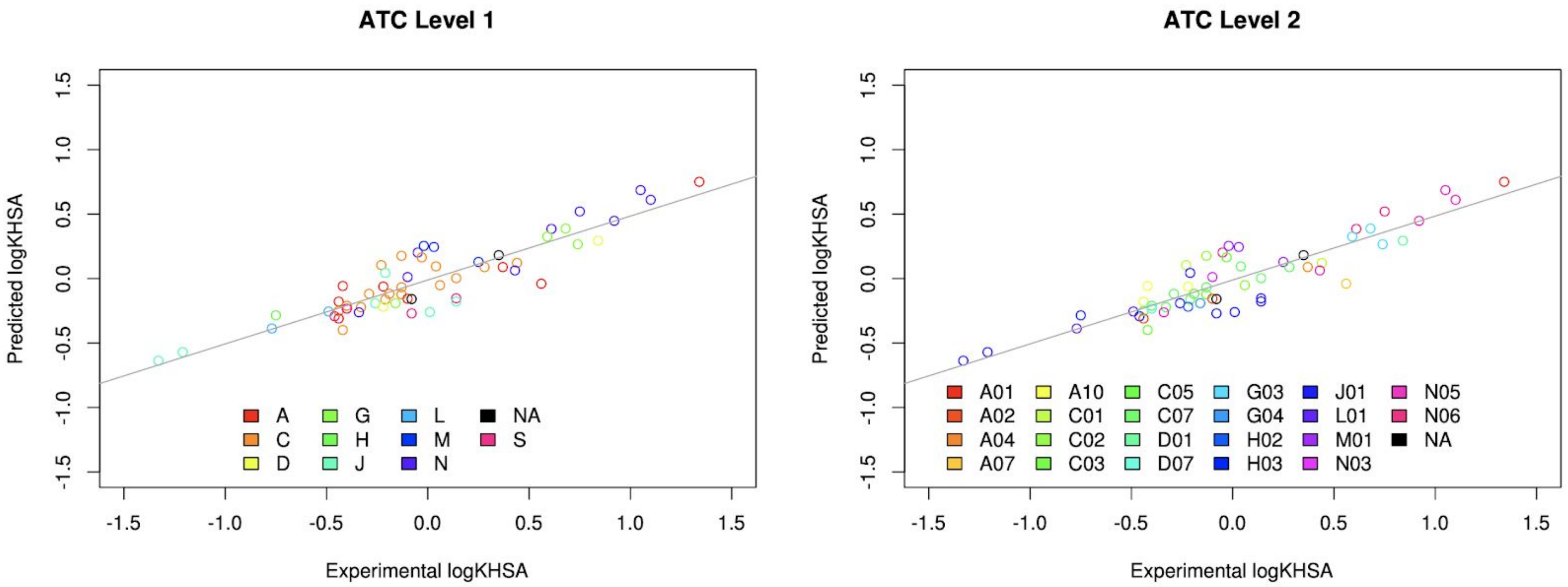

Figure S3: Predicted logK_{HSA} versus experimental logK_{HSA} values or the 59 drugs coloured by ATC code level 1 and level 2
