# Supplementary material for: An integrated quantitative structure and mechanism of action-activity relationship model of human serum albumin binding
Source: J Cheminform. 2019 Jun 6;11:38. doi: 10.1186/s13321-019-0359-2 (PMC6551915; doi:10.1186/s13321-019-0359-2)
Supplement: Supplementary file 8 — Additional file 8. Fig. S5. Boxplot of the predicted logKHSA values of the 799 external compounds coming from CMap dataset grouped by ATC codes level 2. [file 13321_2019_359_MOESM8_ESM.pdf]

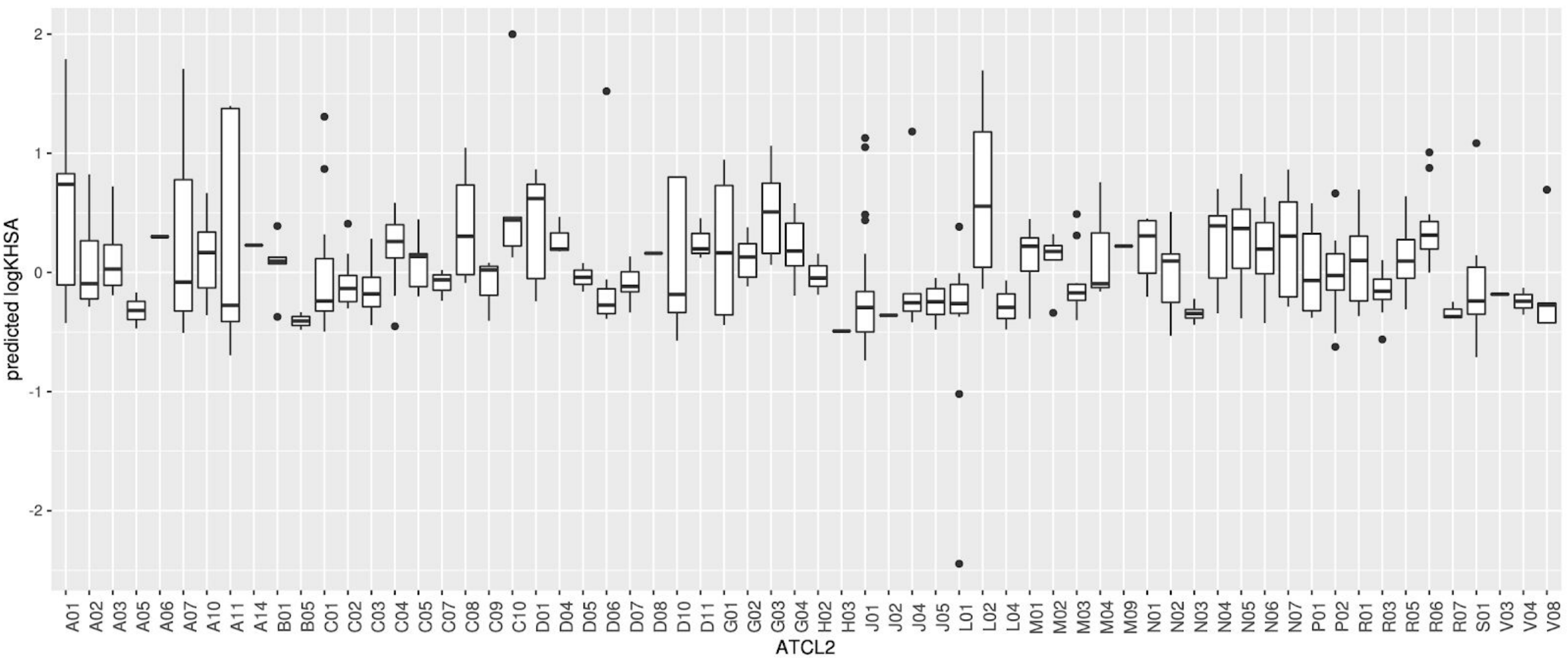

Fig S5: Predicted $logK_{HSA}$ values for the external 799 compounds in the CMap dataset grouped by ATC code level 2.
