# Supplementary material for: An integrated quantitative structure and mechanism of action-activity relationship model of human serum albumin binding
Source: J Cheminform. 2019 Jun 6;11:38. doi: 10.1186/s13321-019-0359-2 (PMC6551915; doi:10.1186/s13321-019-0359-2)
Supplement: Supplementary file 9 — Additional file 9. Fig. S6. Boxplot of the expression values of the three selected genes, grouped by immunosuppressant and their less similar drugs. [file 13321_2019_359_MOESM9_ESM.pdf]

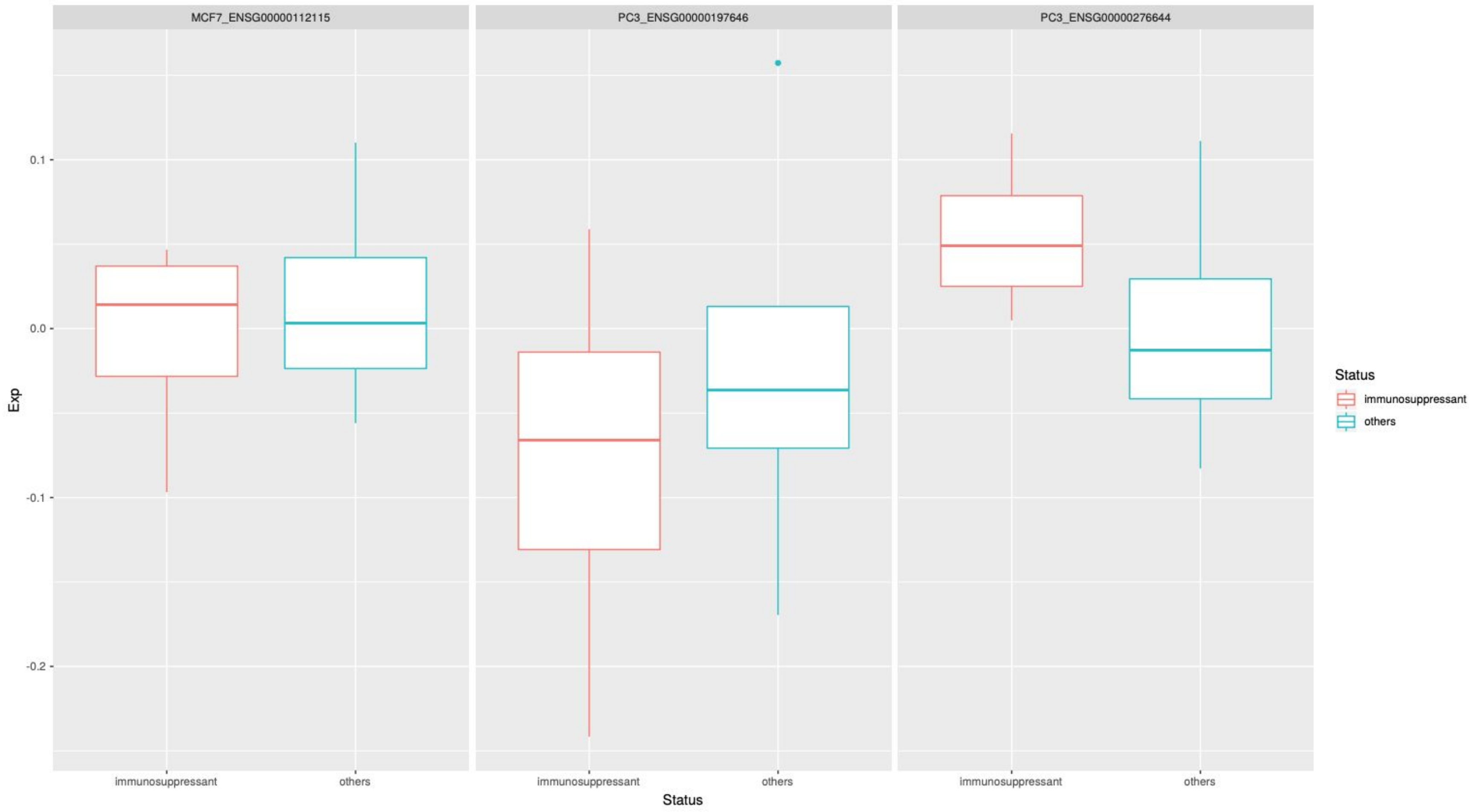

Fig S6: Expression values of the three genes included in the QSAMRt model for immunosuppressant and their most dissimilar drugs in the external set coming from the CMap dataset
